# Deep neural network learning of complex binary sorption equilibria from molecular simulation data†
†Electronic supplementary information (ESI) available: Experimental details for machine learning and Monte Carlo simulation for binary sorption systems investigated, neural network prediction results for equilibrium loadings in systems other than MFI-C5-W, and additional method details for desorption operation optimization. See DOI: 10.1039/c8sc05340e


**DOI:** 10.1039/c8sc05340e

**Published:** 2019-03-18

**Authors:** Yangzesheng Sun, Robert F. DeJaco, J. Ilja Siepmann

**Affiliations:** a Department of Chemistry and Chemical Theory Center , University of Minnesota , 207 Pleasant Street SE , Minneapolis , Minnesota 55455-0431 , USA . Email: siepmann@umn.edu ; Fax: +1 (612) 626-7541 ; Tel: +1 (612) 624-1844; b Department of Chemical Engineering and Materials Science , University of Minnesota , 412 Washington Avenue SE , Minneapolis , Minnesota 55455-0132 , USA

## Abstract

We employed deep neural networks (NNs) as an efficient and intelligent surrogate of molecular simulations for complex sorption equilibria using probabilistic modeling. Canonical (*N*_1_*N*_2_*VT*) Gibbs ensemble Monte Carlo simulations were performed to model a single-stage equilibrium desorptive drying process for (1,4-butanediol or 1,5-pentanediol)/water and 1,5-pentanediol/ethanol from all-silica MFI zeolite and 1,5-pentanediol/water from all-silica LTA zeolite. A multi-task deep NN was trained on the simulation data to predict equilibrium loadings as a function of thermodynamic state variables. The NN accurately reproduces simulation results and is able to obtain a continuous isotherm function. Its predictions can be therefore utilized to facilitate optimization of desorption conditions, which requires a laborious iterative search if undertaken by simulation alone. Furthermore, it learns information about the binary sorption equilibria as hidden layer representations. This allows for application of transfer learning with limited data by fine-tuning a pretrained NN for a different alkanediol/solvent/zeolite system.

## Abstract 2

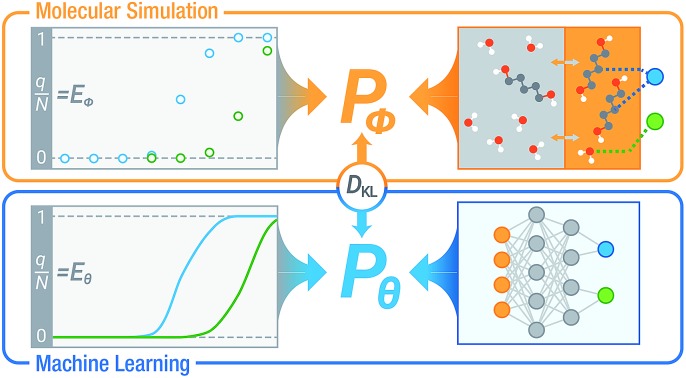
We employed deep neural networks (NNs) as an efficient and intelligent surrogate of molecular simulations for complex sorption equilibria using probabilistic modeling.

## 1 Introduction

Phase and sorption equilibria are ubiquitous, and are necessary for the design of various engineering and industrial operations.^1^–^4^ However, the dimensionality of the *x*_*i*_,*P*,*T*-hypersurface of a mixture of interest increases with each additional component. When components in the mixture are non-ideal such that the mixture behavior cannot be predicted without measurement, this increased dimensionality makes it difficult to accurately describe the entire *x*_*i*_,*P*,*T*-hypersurface.

In addition, binary equilibria are more difficult to measure experimentally than single-component equilibria.^5^ Gmehling *et al.* estimated that less than 2% of the binary mixtures of technical interest have data available for equation of state and excess Gibbs energy models.^6^ To address the lack of experimental data available, molecular simulation has been an effective tool for predicting phase and sorption equilibrium properties in complex thermodynamic systems.^7^–^9^ However, to implement these simulation-based equilibria in modeling of an industrial process, a continuous function is necessary to describe the *x*_*i*_,*P*,*T*-hypersurface (where the thermodynamic state variables *x*_*i*_, *P*, and *T* for an adsorption system denote the mole fraction of component *i* and the pressure of the reservoir phase and the temperature of the system).^10^,^11^

Over the past decade, machine learning has enjoyed unprecedented attention and success in modeling massively complex systems, tasks and behaviors, including image recognition,^12^,^13^ natural language processing,^14^,^15^ and action planning.^16^–^18^ By virtue of fast and accurate evaluation (inference) after being trained, machine learning models are well-suited for the prediction of thermodynamic equilibria. As a predictive thermodynamic modeling approach, machine learning methods have been applied to spin lattices,^19^ supercritical fluids,^20^ multiphase mixtures^21^ and separation processes.^22^–^24^ Moreover, it is noteworthy that a fair number of machine learning models are inspired from and thus closely related to thermodynamic systems.^25^–^27^

Recent achievements of machine learning are mainly attributed to the emergence of deep learning which uses multilayered deep neural networks (NNs) to extract information from input data. Moreover, the features learned by a deep NN are transferable among similar systems or tasks.^28^ As a result, transfer learning can be used to tune a pre-trained deep learning model on a small amount of data for a new problem for faster convergence and lower error. While transfer learning has become a common practice in image recognition and natural language processing,^29^,^30^ predictive thermodynamic modeling can dramatically benefit from the transferability of deep NNs. If a transferable NN is coupled with molecular simulations, properties of a new system can be predicted at a fraction of the computational cost, and the amount of data required to achieve accurate predictions can be much less demanding.

One type of thermodynamic equilibria is adsorption equilibria, where one or more components (adsorbates) are in contact with an adsorbent phase and a reservoir phase. Adsorption isotherms are the most common type of closed-form functions to describe adsorption equilibria at constant temperature.^31^–^39^ Apart from isotherms that mostly describe single-component adsorption, multicomponent adsorption theories^40^–^44^ have been developed for mixture adsorption systems. NNs were also employed in adsorption systems as a replacement of traditional functional isotherms to fit experiments,^45^–^48^ while in this work, transferable deep NNs are developed over molecular simulations of adsorption equilibria to further increase the predictive power.

**Fig. 1:**
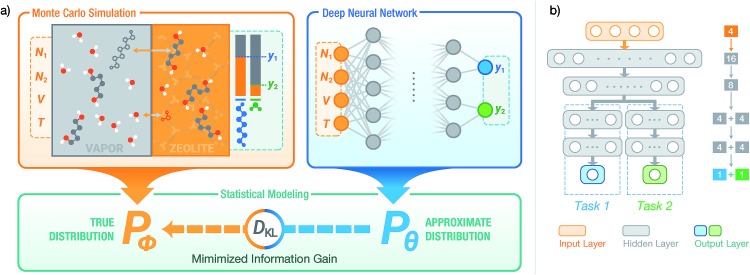
(a) Using a deep NN to approximate the underlying probability distribution obtainable from a Monte Carlo simulation for a binary adsorption system by minimizing the information gain. The NN models the same input and output properties as the simulation. *y*_1_ and *y*_2_ denote the adsorbed fraction of sorbate molecules and *D*_KL_ denotes the Kullback–Leibler divergence. (b) The structure of SorbNet, the multi-task NN used to predict the simulation results. Arrows represent interconnection between two layers and Arabic numerals refer to numbers of units (neurons) for each layer.

Here, we present a modeling workflow that combines molecular simulations with deep NNs to learn the *x*_*i*_,*P*,*T*-hypersurface of complex chemical systems. We consider binary sorption equilibria, where two components (adsorbates) are in contact with an adsorbent phase and a reservoir phase (see Fig. 1). The adsorbing mixtures consist of a linear alkane-α,ω-diol (referred to alkanediol or diol hereafter) and a solvent, either water or ethanol. The adsorbents considered are zeolites, crystalline materials with size-selective pores widely used in industrial applications,^49^–^52^ in the (hydrophobic) all-silica form. These sorption equilibria are necessary for heterogeneous catalysis^53^–^56^ and separation^57^–^61^ applications, and all-silica zeolites can allow for highly-selective separation of diols over water. Prediction of the equilibria of these highly non-ideal mixtures is challenging from only single-component measurements.^62^,^63^ Previously, we have shown that molecular simulations for alkanediol adsorption exhibit great agreement with experiments.^60^ Therefore, the simulations can be trusted to obtain accurate equilibria at conditions difficult to probe experimentally, such as at desorption conditions. At desorption conditions, a large amount of pressure and temperature data is required and is typically unavailable.^64^

To model the *x*_*i*_,*P*,*T*-hypersurface, a machine learning formalism was developed based on underlying principles of statistical thermodynamics, and a deep multi-task NN was trained to the molecular simulation results (see Fig. 1). The NN was then utilized to optimize the temperature for maximum sorbate enrichment of a single-stage equilibrium desorption operation. Furthermore, the transferability of the deep NN was investigated. The information on sorption equilibria for one specific sorbate/framework system learned by the deep NN can be generalized into chemically similar systems through transfer learning, obtaining lower test set errors than retraining the network on the new system.

## 2 Computational methods

### 2.1 Learning formalism for simulation of sorption equilibria

We adopt statistical thermodynamics to establish a machine learning formalism in molecular simulation systems (see Fig. 1a). Machine learning has been applied in predicting the Hamiltonian^65^,^66^ and partition function^67^ to assist simulations. Recently, transition matrix Monte Carlo methods have been utilized to obtain the partition function and the *P*,*T* surface for unary CH_4_, CO_2_, and C_3_H_8_ adsorption,^68^ but the computational complexity of this approach increases as power law with the number of components. In the present work, the focus is on directly predicting the observed properties akin to the simulation results without predicting the partition function. In molecular simulation, a desired thermodynamic property is measured as the expectation value of its corresponding thermodynamic variable in a statistical ensemble obeying a set of thermodynamic constraints. Fundamentally, the simulation system can be viewed as a probabilistic model where the distribution of thermodynamic variables is dependent on the configuration of particles in the system and is implicitly parametrized by the interactions of the particles. When performing simulations in a given ensemble, this implicit probability distribution is conditioned on its thermodynamic constraints. For example, let *y* be a thermodynamic variable in a system *φ*, the corresponding measured value of property *Y* in an *NVT* ensemble simulation is*Y* = 𝔼[*y*]; *y* ∼ *P*_*φ*_(*y*|*N*,*V*,*T*)where the tilde sign denotes that the variable observes a probability distribution. Due to the massive number of degrees of freedom in a microscopic system, it is computationally expensive to perform direct inference from distribution *P*_*φ*_ using a Monte Carlo algorithm to predict the desired properties. Therefore, a NN parametrized by a set of weights *θ* can be used to approximate the actual thermodynamic system *φ*. Our approximate inference approach is analogous to the neural variational inference method,^69^ while the difference is that in the molecular simulation case it is possible to sample from the intractable distribution *P*_*φ*_ despite some cost. By training the NN using information about *P*_*φ*_ from simulation, the distribution learned by NN *P*_*θ*_ is expected to reproduce the simulation outputs related to conditional probabilities, *i.e.*, *P*_*θ*_(*y*|*N*,*V*,*T*) ≈ *P*_*φ*_ (*y*|*N*,*V*,*T*) in an *NVT* ensemble. In this case, the NN would be able to predict the simulated quantity as *Y* ≈ *Ŷ* = 𝔼_*y*∼*P*_*θ*__[*y*|*N*,*V*,*T*].

Particularly, we focus on the loading of each component in a zeolite adsorption system, namely the amount of each component adsorbed in the zeolite phase when it equilibrates with a reservoir phase. In a multicomponent Gibbs ensemble Monte Carlo (GEMC) simulation, the ensemble-averaged adsorption loading of the *i*th component, *q*_*i*_, is directly measured from the number of molecules in the zeolite phase,*q*_*i*_(**N**,*V*,*T*) = 𝔼_*z*_*i*_∼*P*_*φ*__[*z*_*i*_|**N**,*V*,*T*]where *z*_*i*_ is the (fluctuating) number of *i*th-component molecules in zeolite phase and **N** = (*N*_1_, *N*_2_,···,*N*_*k*_) is a vector of total numbers of all components. Then, approximate inference is performed by modeling *P*_*θ*_ as a more tractable closed-form distribution. Specifically, the conditional approximate distribution *P*_*θ*_(*z*_*i*_|**N**,*V*,*T*) is modeled as a binomial distribution,
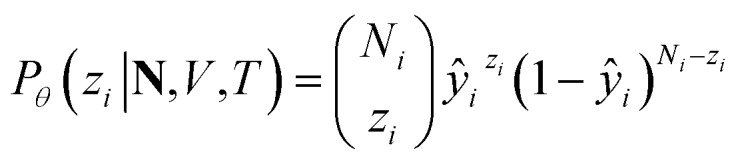
where *ŷ*_*i*_ = *f*_*θ*_(**N**,*V*,*T*) is the binomial coefficient given by the NN. The implication of the binomial predicted distribution is that at each state point, it learns an equivalence between the real adsorption system and an ideal non-interacting adsorption system where particles have a uniform probability to be adsorbed. Information about the simulation system has been lost through this approximation, hence the objective of the learning algorithm is to minimize the information gain from *P*_*θ*_ to *P*_*φ*_. The Kullback–Leibler (KL) divergence^70^ is used as the metric of information gain to train the NN,
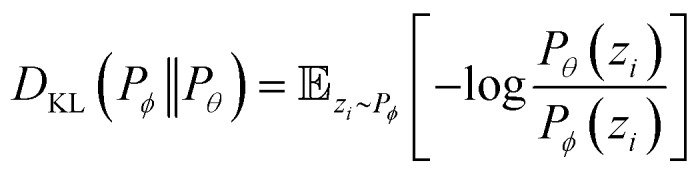


At a given input state (**N**,*V*,*T*), the conditional KL divergence can be evaluated using the binomial equation of *P*_*θ*_(*y*|**N**,*V*,*T*),
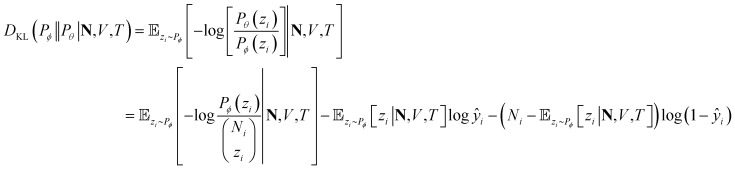
using the relationship *q*_*i*_(**N**,*V*,*T*) = 𝔼_*z*_*i*_∼*P*_*φ*__[*z*_*i*_|**N**,*V*,*T*] gives
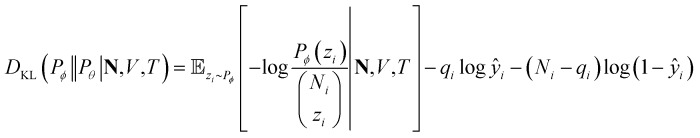
the first term in the KL divergence is independent of the predicted distribution *P*_*θ*_, so the loss function for an input (**N**,*V*,*T*) for minimization is*l*(*q*_*i*_,*ŷ*_*i*_) = –*q*_*i*_ log *ŷ*_*i*_*–* (*N*_*i*_ – *q*_*i*_)log(1 – *ŷ*_*i*_)therefore, the total loss of the learning algorithm for all components on all inputs in the training set is
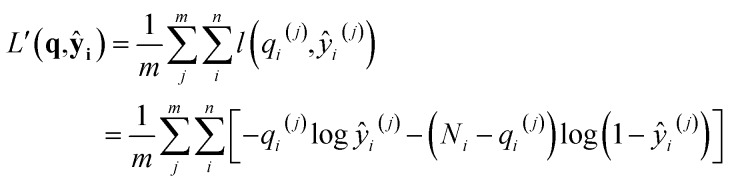
where superscripts denote training examples and *m* is the total number of training data. Assuming that the finite size effect of simulation on the expectation value can be ignored, the prediction of the NN can be applied to an equivalent system of any size since the NN model does not account for variance. Therefore, the input state (**N**,*V*,*T*) was normalized into a set of intensive (relative) properties (**n**,*v*,*T*) = (**N**/*N*_z_,*V*/*N*_z_,*T*) where *N*_z_ is the number of zeolite unit cells in the system. Also, the ensemble averaged equilibrium loading is normalized into the range of [0,1] as *y*_*i*_ = *q*_*i*_/*N*_*i*_. After normalization, the loss function of the NN which predicts the equilibrium loading of the *i*th component becomes
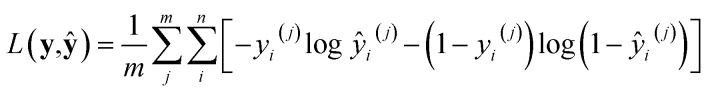
with *ŷ* = *f*_*θ*_(**n**,*v*,*T*). Interestingly, *L*(**y**,**ŷ**) coincides with the cross-entropy loss function in a binary classification problem, which makes the learning algorithm implemented more readily.

### 2.2 Multi-task learning of binary desorption simulation

We apply the learning formalism described in the preceding section to predict the equilibrium loading of components in a binary GEMC simulation system. Here, we are interested in modeling the desorptive “drying” of an adsorbate mixture from a loaded framework that is part of a three-stage process consisting of adsorption from a solution phase, partial desorption of the solvent (drying) into a vapor phase, and desorption of the desirable product (see Fig. S1 in the ESI†).^71^,^72^ Specifically, all-silica zeolites have been shown to be exceedingly selective adsorbents for the separation of alcohols from aqueous solution.^60^,^63^,^73^ For GEMC simulations of the desorptive drying process, specific initial loadings were taken from prior GEMC simulations of the solution-phase adsorption at *T* = 323 K (see Table S2 in the ESI†). These prior GEMC simulations were performed with an explicit solution phase because of the highly non-ideal behavior of these solutions. For the adsorption points considered here, the concentration of the alkanediols varied by up to three orders of magnitude, whereas the concentration of the solvent varied by less than a factor of 2 (*i.e.*, the chemical potential of the solvent is nearly constant over a large part of the concentration range for adsorption, see Table S2 in the ESI†). Desorptive drying of the loaded zeolite usually occurs into a low-pressure vapor phase at a higher temperature. In the desorption simulation, the loaded zeolite phase is equilibrated with an empty vapor phase at a constant volume. Since adsorption occurs from a solution phase at low temperature and atmospheric pressure, whereas desorptive drying of the mixture occurs into a vapor phase at high temperature and low pressure (and the desorption of the product occurs at even higher temperature also into a vapor phase), these processes are distinct and do not reflect a hysteresis loop.^74^ Finding suitable conditions for desorptive drying is challenging because of the desire to desorb nearly all of the solvent while desorbing nearly none of the product.

The GEMC set-up allows one to probe desorption of a zeolite loaded with a specific number of molecules, whereas many grand canonical Monte Carlo simulations would be needed to find the set of chemical potentials that corresponds to this specific desorptive drying scenario. The loadings of both components in the zeolite phase are measured after equilibration of the desorption process. Two types of diols, butane-1,4-diol (C4) or pentane-1,5-diol (C5) were used, water (W) or ethanol (E) were used as the solvent, and the chosen adsorbents were all-silica zeolites of the framework types MFI and LTA. The zeolite–diol–solvent combinations for which simulation data were obtained are MFI-C5-W, MFI-C5-E, MFI-C4-W and LTA-C5-W. A multi-task learning model was employed to simultaneously predict the loadings of both diol and solvent to account for the behavior of both components,{*ŷ*_1_,*ŷ*_2_} = *f*_*θ*_(**n**,*v*,*T*)where **n** = (*n*_1_,*n*_2_) are the relative total numbers of the two components as initial loadings. A branched multi-task structure was used to construct the deep NN for loading prediction where the prediction for both components share the lower (closer to input) layers and have independent higher layers. A schematic diagram for the multi-task network is shown in Fig. 1b. The activation functions of the NNs are chosen such that the NN is able to produce physically reasonable outputs. The ELU function^75^ used throughout hidden layers is continuously differentiable so that the loading surface predicted by the NN always has continuous derivatives. The predicted fractional loadings of both components are produced by the sigmoid function in the output layer, which ensures that the fractional loadings always satisfies 0 ≤ *ŷi* ≤ 1, *i.e.*, the number of molecules of any type in any phase cannot become negative or exceed the total number of molecules of this type. Through sharing the lower layers among different prediction tasks, the lower layers of a multi-task network are able to learn about the information of the whole system as well as reduce the tendency of overfitting.^76^ This NN structure is referred to as ‘SorbNet’ for convenience. The SorbNet code and the datasets used in the current work are available *via* Github (see ESI†).

Simulations for each desorption system were performed at 16 temperatures (343 K to 493 K in steps of 10 K), 16 logarithmically-spaced vapor-phase volumes, and 4 initial sorbate loadings, and the results were collected for 32 independent simulations at each set of thermodynamic state variables. This gives 1024 state points and 32 768 simulation data entries for each system, and 131 072 simulations for the four systems. The details of the molecular simulation are reported in the ESI (Section S2†).

The reasons why multiple independent simulations were performed at a single state point are that carrying out independent simulations reduces the wall clock time needed to obtain results of a desirable statistical significance, and that the statistical uncertainty of the simulation prediction can be estimated from independent simulations in a straightforward manner. Taking subsets of the independent simulations to train separate NNs can provide a path to uncertainty estimation. To capture the uncertainty among independent simulations, the bagging method for ensemble learning was used to obtain the mean and uncertainty for NN predictions.^77^,^78^ Using 32 SorbNets each trained against data from one independent simulation at different state points, the mean and standard deviation from their 32 predictions can be compared with simulation statistics. It is found that the standard deviation from the ensemble learning reflects the uncertainties of the simulation data (see Fig. S4 in the ESI†). Using data from the 32 independent simulations enables more facile training of SorbNet compared to using the mean from the independent simulations.

**Fig. 2:**
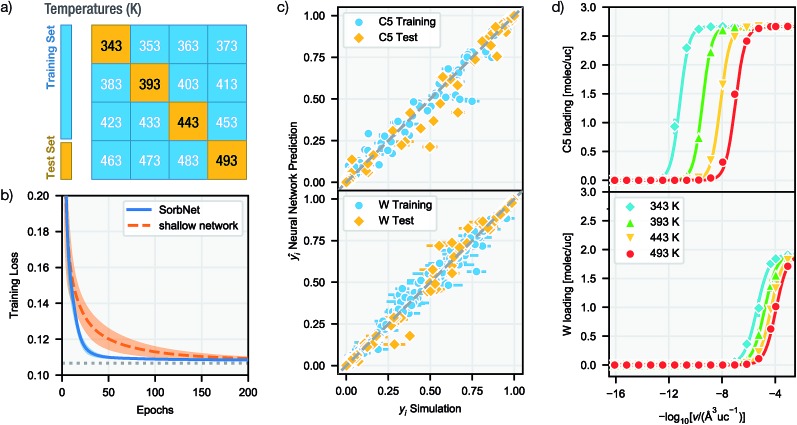
Deep NN learning of the MFI-C5-W desorption simulation system. (a) The training/test temperature allocation. (b) Learning curves of SorbNet and a shallow NN with the same parameter complexity. The dotted horizontal line denote minimum training loss attainable by predicting the averaged result for each group of independent simulations. (c) Scatter plot of SorbNet predictions for C5 (top) and W (bottom) fractional loadings for training and test temperatures *versus* fractional loadings obtained from simulations. (d) Loading-volume sorption isotherms of the MFI-C5-W system at test-set temperatures and an initial loading of C5 : W = 2.67 : 1.92 (molec/uc). Symbols denote simulation data and lines denote NN predictions. Abbreviations: molec – molecule; uc – unit cell.

The training–validation (test) set split for simulation data was performed according to the temperature of data points. In molecular simulation workflows, a whole isotherm sequence at a specific temperature and different molecule numbers (or pressures) is usually determined instead of data from random state points to probe the adsorption behavior. Based on this convention all data at 4 out of 16 temperatures were held out to construct the validation (test) set (see Fig. 2a).

Transfer learning experiments were also carried out on the NN model. First a NN model was trained using the training set described above. Then the pre-trained NN was transferred on either the training set of a different sorption system or a much smaller transfer set constructed by picking 1 out of 16 temperatures of the simulation data.

## 3 Results and discussion

### 3.1 Neural network prediction of simulation results

Trained on all simulation results for 12 temperatures for 500 epochs (cycles), the deep NN was able to achieve accurate predictions at both training and test temperatures with an accuracy comparable to the precision of simulation results. Fig. 2c gives a comparison between simulation results and NN predictions for training and test sets in each sorption system. The predictions of SorbNet for the binary diol/water mixtures are very encouraging because standard thermodynamic models, such as the ideal adsorbed solution theory^40^ or the competitive Langmuir model,^32^ would not be able to describe the binary loading because neat water would show negligible loading at all conditions considered here,^79^ and only the hydrogen-bonding with diols leads to co-adsorption of water.^60^,^63^

The mean square errors (MSE) for the training and test sets in each sorption system are listed in Table 1. The simulation variance for a system is given as the average variance of the normalized equilibrium loading **y** for 32 independent simulations at all 1024 state points in the system. In fitting the training set data, the SorbNet achieved an accuracy comparable to simulation precision with the mean square error at around twice of the averaged variance for the simulation result. When predicting the unseen test set data, the SorbNet also maintained an accuracy level at the same magnitude as the simulation precision. Although the predictions from MC simulations can be made more precise by running longer simulations, the uncertainties arising from force field parameterization^79^ and framework structure^60^ will not be affected by doing so. On the other hand, while the prediction error of a NN can be decreased by increasing the number of neurons,^80^ the number of NN parameters must be kept smaller than the number of data points in the training set. In the present work, we found that increasing the complexity of the NN did not dramatically improve the predictions.

**Table 1:** NN training and prediction for desorption simulation results

Sorption system	Model	Training MSE^*a*^ (×10^–4^)	Test MSE^*a*^ (×10^–4^)	Simulation variance (×10^–4^)
MFI-C5-W	**SorbNet**	**3.8 ± 0.5**	**9.3 ± 0.9**	2.8
	Shallow	4.3 ± 0.2	9.4 ± 0.4	
MFI-C4-W	**SorbNet**	**2.5 ± 0.4**	**4.5 ± 0.7**	1.9
	Shallow	4.1 ± 0.5	7.2 ± 1.1	
MFI-C5-E	**SorbNet**	**1.9 ± 0.4**	**5.6 ± 0.9**	0.7
	Shallow	2.7 ± 0.3	7.5 ± 0.7	
LTA-C5-W	**SorbNet**	**2.8 ± 0.6**	**7.6 ± 1.6**	1.4
	Shallow	3.8 ± 0.2	9.6 ± 0.5	

^*a*^Mean square errors were evaluated using the averaged simulation result as the true value. Standard deviations were measured from 8 training runs.

To justify the design of SorbNet structure, the performance of SorbNet was compared against a shallow NN with the same number of parameters since a shallow network is already able to approximate any continuous function due to the universal approximation theorem.^80^Fig. 2b shows the training curves of SorbNet and the shallow network over the first 200 epochs. The shallow network learned at a lower efficiency on simulation data evidenced by a much slower convergence. The inefficiency of a shallow network is likely to be a result from strong correlation among hidden layer units, as 48 hidden layer representations are mapped from only 4 input features. Moreover, the shallow network gave a slightly higher training and test error after convergence. Those observations prove that a deep NN is able to achieve superior performance to a shallow NN albeit the latter already has enough theoretical prediction power.

Since NNs perform significantly faster evaluations than simulations, it is possible to interpolate a numerically continuous isotherm curve using NN predictions. Fig. 2d shows the interpolated isotherm curves by SorbNet at one representative sorbate composition in the test set, and NN prediction agrees well with the simulation results.

### 3.2 Application of SorbNet to optimize process conditions

**Fig. 3:**
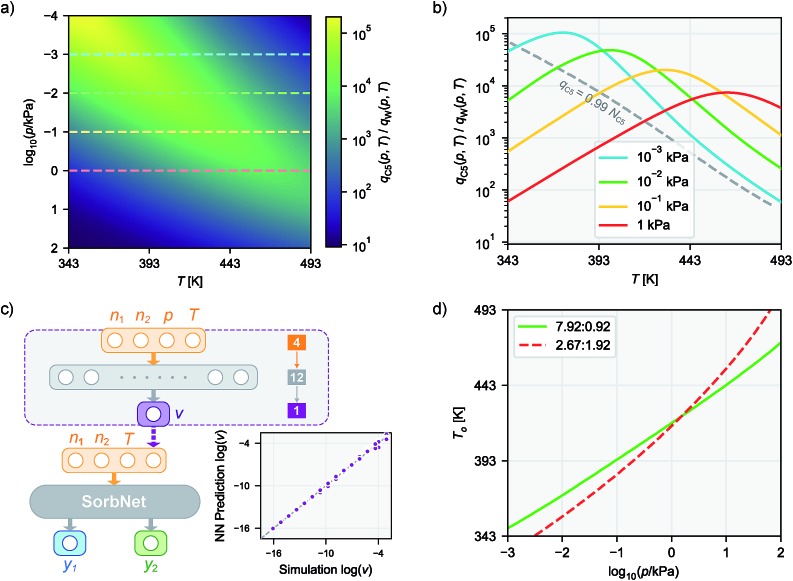
Optimizing the temperature of isobaric desorption process using NN loading surfaces in the MFI-C5-W system. (a) The *p*–*T* heatmap for the adsorbed C5/W molar ratio at initial loading C5 : W = 7.92 : 0.92 (molec/uc) predicted by SorbNet network. Horizontal dashed lines denote isobaric desorption. (b) C5/W molar ratio as a function of temperature at different operation pressures at initial loading C5 : W = 7.92 : 0.92 (molec/uc) predicted by NN. The dashed line represents the C5/W molar ratio as a function of temperature at *q*_C5_ = 0.99*N*_C5_. The intersection between an isobaric curve and the dashed line corresponds to the optimal temperature. (c) NN mapping of total pressure to relative reservoir volume. The inset illustrates the accuracy of the *p*–*v* mapping for the MFI-C5-W system. (d) The dependence on pressure for the optimal temperature *T*_o_ at two different C5/W initial loadings.

We applied the interpolation ability of SorbNet to find the optimal temperature for a single-stage, equilibrium-based desorption process. In this desorptive-drying process step, the loaded zeolite is subjected to a pressure and temperature swing in order to remove the solvent impurities from the alkanediol product. At a given pressure, a low temperature may not remove enough of the solvent, while a high temperature will remove both the solvent and the diol. Therefore, it is likely that there exists an optimal temperature for this task. Mathematically, the loadings of diol and solvent *q*_*i*_ (*p*,*T*) are regarded as functions of temperature and pressure given the initial loadings *N*_*i*_ in the operation setting, and the optimal temperature is obtained as
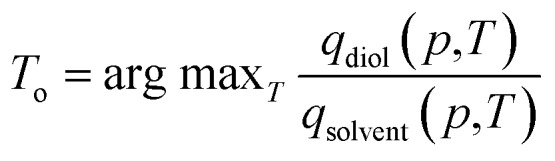
where argmax *f* (·) gives the parameter which maximizes the value of the objective function *f* (·). A constraint that *q*_diol_ (*p*,*T*) ≥ 0.99*N*_diol_ is also imposed to preserve the diol loading in the zeolite (and overall yield of the three-stage process). This choice of optimal temperature makes a subsequent desorption step at higher temperature/lower pressure yield an extremely high-purity diol product without compromising diol recovery. It should be noted that commonly used ideal adsorbed solution theory^40^ would not be suitable for this optimization task because it would predict almost complete desorption of water at all process conditions due to the low unary uptake of water in hydrophobic zeolites that does not account for co-adsorption induced by alcohols.^60^,^63^ For the MFI-C5-W system, the optimal temperature always occurs at the constraint boundary, therefore the optimization can be alternatively done by searching for the root of *q*_diol_ (*p*,*T*) ≥ 0.99*N*_diol_ (Fig. 3b).

To obtain an adsorption hypersurface in terms of pressure, the total vapor pressure was calculated using vapor phase densities from simulation assuming an ideal gas in the vapor phase. Another NN (*p*–*v* network) was used to map the total vapor pressure *p* to relative vapor phase volume *v* by taking a state (*n*_1_,*n*_2_,*p*,*T*) as input. Since it is a trivial prediction task, a shallow network much smaller than SorbNet was adopted and its output was coupled with SorbNet to predict the equilibrium loadings (Fig. 3c). Subsequently, an isobaric adsorption curve with varying temperatures is produced. A 2D heatmap for the diol–solvent molar ratio in the zeolite phase is shown in Fig. 3a. For an isobaric equilibrium operation (a horizontal line), a temperature with the maximum molar ratio exists in the heatmap, and the optimal temperatures are found at the constraint boundary for the MFI-C5-W system (Fig. 3b). Using two representative initial loadings, the optimal temperature as a function of operation pressure was calculated by maximum searching in 0.2 K intervals from 343 K to 493 K (Fig. 3d). For an initial composition with a lower diol–solvent ratio, the slope of *T*_o_*versus* log-pressure is higher, indicating that it is more difficult to maintain high recovery (*i.e.*, 99% fractional diol loading) when conducting the isobaric desorption operation. This desorptive-drying optimization problem would be much more difficult using molecular simulations and traditional isotherm fitting. If the optimization was to be undertaken by simulations alone, a sequence of desorption simulations would need to be conducted iteratively to search for the optimal temperature following the bisection method. If adsorption isotherm modeling was to be used, the equilibrium vapor phase composition as well as the partial pressures are not known beforehand, so it would be difficult to fit multicomponent isotherms as they almost always require the partial pressures of all components.

### 3.3 Transfer learning of SorbNet to new sorption systems

Due to the nature of deep NNs, we hope that the SorbNet can learn information about sorption equilibria in a manner analogous to humans. We characterize the hidden layer features learned from desorption simulations by application of transfer learning. When trained on a given task, a deep NN is able to learn more general features which can be transferred to learning similar tasks.^28^ In this context, it is likely that the feature that the SorbNet has learned on a sorption system can be transferred to other chemically similar systems.

Since the NN always predicts the same output property while the microscopic configurations vary by different systems at the same state point, the branched higher layers of SorbNet were transferred as different systems share output semantics.^81^ As an intuition of its multi-task design, we expect that the lower shared layers of SorbNet extract information about a particular sorption system and the higher branched layers calculate the adsorption loadings from the extracted information and work in a system-independent manner. Such information can be as simple as adsorption decreases with temperature, and can also be more complex thus difficult to observe. This also echos conventional thermodynamic modeling in that coefficients determining how state variables are related to each other contain information about the system, such as the critical point in the Peng–Robinson equation of state.^82^ The hypothesis that the higher layer weights are general to different sorption systems was proved by pre-training the SorbNet structure on one adsorption system, keeping the pre-trained weights of branched layers while reinitializing the lower layers and retraining it on another system with different zeolite or sorbates. The transfer learning results to other sorption systems with the branched layer weights either fixed or trainable are shown in Table 2.

**Table 2:** Transfer learning and fine-tuning of SorbNet pre-trained on MFI-C5-W system

Sorption system	Initialization	Branched layers	Training MSE^*a*^ (×10^–4^)	Test MSE^*a*^ (×10^–4^)
MFI-C4-W	Pre-trained	Fixed	2.9 ± 0.6	5.0 ± 1.2
	Pre-trained	Trainable	2.6 ± 0.5	4.6 ± 1.1
	**Random**	**Trainable**	**2.5 ± 0.4**	**4.5 ± 0.7**
	Random	Fixed	105 ± 6	113 ± 8
MFI-C5-E	Pre-trained	Fixed	2.5 ± 0.5	6.4 ± 1.4
	**Pre-trained**	**Trainable**	**1.6 ± 0.6**	**4.4 ± 1.4**
	Random	Trainable	1.9 ± 0.4	5.6 ± 0.9
	Random	Fixed	150 ± 3	155 ± 5
LTA-C5-W	Pre-trained	Fixed	3.5 ± 0.3	9.2 ± 0.7
	**Pre-trained**	**Trainable**	**2.8 ± 0.5**	**7.5 ± 1.5**
	Random	Trainable	2.8 ± 0.6	7.6 ± 1.6
	Random	Fixed	(2.0 ± 1.7) × 10^2^	(2.2 ± 1.7) × 10^2^

^*a*^Standard deviations were measured from 8 training runs. 8 models independently pre-trained on MFI-C5-W system used as initialization in transfer learning experiments.

Compared with training a network from scratch on the new system, retraining the shared lower layers with fixed branched layers resulted in a slightly higher error yet generally at the magnitude on par with a new network. When the branched layers are further allowed to be fine-tuned, transfer learning achieves statistically indistinguishable performance from training a new network (Table 2). However, an alternative explanation for those results is that the lower layers have already had the enough capacity to accomplish the entire prediction task, in which case the information in the higher layers are irrelevant. To inspect this possibility, another SorbNet structure was also trained on each sorption system with its higher layers fixed at randomly initialized weights. In machine learning practice, a way to probe whether the NN is overcomplicated for the task, is to check whether it even fits the data with random labels. If the lower layers already fit random outputs given by the initial weights of the higher layers, it would be irrelevant whether the branched layers have extracted any useful features. As is shown in Table 2, training the lower layers against random higher layer weights resulted in considerably higher errors. Conclusively, the higher branched layers of SorbNet indeed play a role in predicting sorption loading from features extracted by lower layers, and are transferable among different sorption systems.

**Fig. 4:**
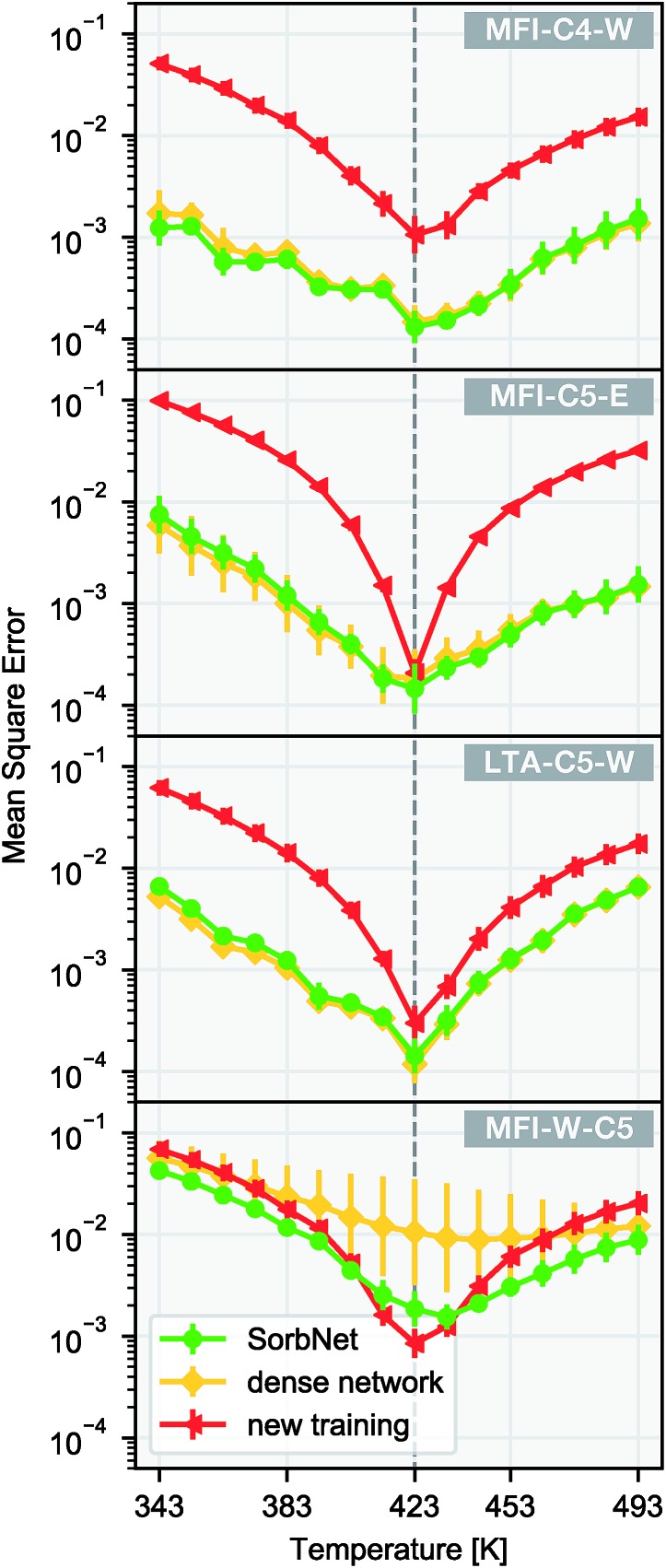
Temperature dependence for sorption loading using transfer learning. A SorbNet structure and a dense network were pre-trained on the 12-temperature training set of the MFI-C5-W system, and then the first two layers were fine-tuned on the 1-temperature transfer set of MFI-C4-W, MFI-C5-E, LTA-C5-W, and the sorbent-switched MFI-C5-W systems. The vertical dashed line indicates the training temperature (423 K). Error bars are calculated as standard deviations of 8 training runs.

We utilize the transferability of the SorbNet in the prediction of temperature dependence for a sorption simulation system with the data at only 1 temperature. Since the lower layers also encode potentially useful information about the sorption system, we kept the lower layer weights instead of reinitializing them when performing transfer learning. In this transfer application, the transfer performance of SorbNet was compared with another deep NN with the same number of hidden layers and a very similar parameter complexity. Its difference with the SorbNet is that it does not have branches and all units are interconnected between layers and is referred to as ‘dense network’ for convenience. Both SorbNet and the dense network were first pretrained on MFI-C5-W system and then fine-tuned on the first two layers in the 1-temperature transfer set for other systems. The NNs were only pre-trained for 200 epochs to prevent overfitting. In addition, a newly initialized SorbNet structure was trained on the transfer set as a baseline for each system. The results for temperature dependence prediction on the transfer set are shown in Fig. 4.

Among all target sorption systems for transfer, the pre-trained SorbNet consistently outperformed the newly-trained baseline in terms of errors at most testing temperatures. SorbNet did not exhibit statistically poorer performance than the dense network, indicating that full connection between every two layers is not necessary. Apart from transferring to different sorption systems, another transfer learning task was also created where the identities of the alkanediol and the solvent in the pre-training system were switched by swapping their corresponding initial and equilibrium loading variables (MFI-W-C5). The intention to create this transfer task is that branches of SorbNet are supposed not to discriminate between stronger and weaker interacting adsorbates, and the split in the SorbNet design encourages the higher-layer features to be general for any sorbate. Since the two branches work independently, they only differ in recognizing which features are relevant to the first or second sorbate in their input. Therefore, the branches are trained to only distinguish between the ‘first’ and the ‘second’ sorbate in the data supplied. In the sorbate-switched system, the SorbNet maintained a mostly lower test error than baseline, while the dense network had a substantially poorer performance and gave a much more unstable training result (Fig. 4d). This can be explained by the tendency of the dense network to ‘remember’ which sorbate binds more strongly with the zeolite in its higher layers, implying the positive effect of a branched structure design of SorbNet.

Another potential benefit of SorbNet's branched structure to its transferability is the prevention of complex co-adaptation by network pruning. Co-adaptation in NNs refers to the phenomenon that the response (output) of different neurons to the training data are always strongly correlated, typically involving the cancellation of excessively large values produced by neurons. Therefore, when the network operates on the test set or is transferred to a different dataset, such correlation may be broken (large values cannot be cancelled), leading to severe overfitting. One common way to prevent co-adaptation is to prune the network structure. With connections between neurons reduced, the neurons are less likely to be tightly correlated with each other. Random pruning of the NN is one of the key ideas in the well-known Dropout method,^83^ while the higher layers of SorbNet are intentionally (rather than randomly) pruned into two separate branches, which also improves transferability of the network.

**Fig. 5:**
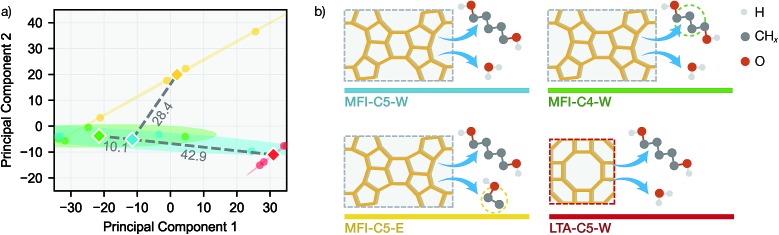
Similarities between sorption systems for NN learning. (a) Principal component analysis on the *q*(*V*,*T*) adsorption patterns for each simulation system. Circles denote individual adsorption patterns and diamonds denote the centroid for each simulation system in the principal component space. Shaded ellipses correspond to 95% confidence interval from covariance matrix. Grey dashed lines denote the distances between centroids with numerical values labelled above each line. (b) Graphic illustration of the sorption systems. Dashed rectangles denote a zeolite unit cell projected along (010) direction and yellow wireframes indicate the network of Si atoms.

To further investigate the difference in transfer performance among new sorption systems, we evaluated the similarities between simulation systems using data-driven methods. For each simulation system and desorption initial loading, the equilibrium loadings were measured at exactly the same temperatures and vapor volumes, allowing us to directly compare the sorption patterns. Principal component analysis (PCA) was performed on the 16 *q*(*V*,*T*) adsorption patterns (4 loadings each for 4 systems) in the full simulation dataset (see Fig. 5a).

Also we measured the distance between the mean over 4 loadings (centroid) of MFI-C5-W system and those of other 3 systems in the 2D principal component space. Interestingly, the PCA similarities between simulation systems agrees well with chemical intuition (see Fig. 5b). With only the alkanediol different by one CH_2_ unit, the adsorption pattern of MFI-C4-W is very similar to that of MFI-C5-W. The MFI-C5-E system uses an organic solvent instead of water, thus having a lower similarity due to fairly different solvent–zeolite interactions. For the LTA-C5-W system, the pore structure of a new zeolite makes it also less similar system to MFI-C5-W among the zeolite and sorbate combinations. Comparing the system similarities with the transfer learning results in Fig. 4, SorbNet exhibits a higher generalization error when transferring to a less similar system at a temperature far from the transfer set, since the information learned on the MFI-C5-W system is applied less effectively when the zeolite or sorbate becomes more different.

It should be emphasized that SorbNet operates only on the thermodynamic state variables (*n*_1_,*n*_2_,*v*,*T*) instead of attributes of the zeolites and sorbates, while this does not prevent it from being transferable among similar sorption systems. More specifically, ‘transfer’ in machine learning refers to applying already-trained features (weights) onto a different dataset or task, and those weights are either fixed or further tuned against the new task. In thermodynamic modeling, this is analogous to assuming that two compounds similar in shape and polarity possess similarities in their fluid phase equilibria and properties, *i.e.*, the principle of corresponding states. Therefore, even without supplying descriptors about the sorbates and the sorbent (*e.g.*, kinetic diameter or limiting pore diameter), SorbNet is transferable to similar sorption systems when limited data for the new system are provided. Nevertheless, SorbNet is likely to transfer very poorly if the sorbates or sorbent are drastically different. Conversely, if descriptors about the sorption system are explicitly contained in the input data, prediction of different sorption systems would be usually considered as generalization instead of transfer learning. This is because, in this hypothesized setting, the NN was intended to fit to different sorption systems already during training. If it is sufficiently predictive, then the exact same set of weights can still be used when the descriptor values for the sorption system vary. In this case, the NN is always performing the same task, and is essentially generalizing from the training set to the test set.

## 4 Conclusion

We developed a modeling workflow that combines molecular simulations with machine learning to predict the sorption behavior of complex chemical systems. Using a deep NN as the machine learning model, we predicted accurately not only the sorption loading results from simulation parameters, but also demonstrated an optimization application which would be very challenging without the assistance of the NN. Moreover, we managed to perform transfer learning to apply machine knowledge learned from one sorption system to systems with different guest–host identities.

The learning formalism proposed focuses on approximating the expectation value for a thermodynamic variable. However, it is possible to extend this approach to the joint learning of multiple probabilistic metrics for a thermodynamic system, such as learning both expectation and variance. In the experiments of transfer learning, the selection of sorption systems was limited within the variation of zeolites and sorbate molecules. Since SorbNet employs a multi-task architecture, it would be of great interest to expand the scope of transfer learning to pre-training on the unary sorption systems for each sorbate and transfer on the corresponding binary system.

A few limitations of SorbNet are emphasized next. One major limitation is that SorbNet is trained on and predicts sorption data for one specific combination of adsorbates and porous framework. Therefore, to predict another sorption system (changing adsorbates and/or framework), some information on the new system is required to retrain the NN using transfer learning. This makes SorbNet inefficient for high-throughput screening where predictions across a large number of porous frameworks are desired for the same state point (partial pressures of adsorbates and temperature). For the same reasons, the SorbNet predictions would hold when changes in framework structure (*e.g.*, including framework flexibility) and force field parameters do not yield significant changes in the simulation outcomes (*i.e.*, for SorbNet, a change in force field parameters is equivalent to changing the adsorbate/framework combination) but, again, some limited new simulation data and re-training through transfer learning would improve accuracy of the SorbNet predictions. Hence, it would be interesting to include representations of diverse porous frameworks and sorbates into the machine learning system so that the NN does not need to be retrained upon changing the sorption system. Another limitation is that the predictions of SorbNet rely more heavily on training data than on the physical principles underlying adsorption. As a result, the NN is prone to yield thermodynamically inconsistent data when (derivative) properties, such as the heat of adsorption, are calculated from the NN predictions. This could be improved by embedding physical constraints as regularization of the NN.^84^ In addition, SorbNet cannot make predictions for the same set of adsorbates and framework for state points far outside the training set (*e.g.*, the diol/solvent adsorption from the liquid phase at relatively low temperature is too far removed from the desorptive drying conditions).

Our work provides a new avenue into applying machine learning in conjunction with molecular simulations for modeling sorption equilibria. Machine learning has revolutionized a large number of computational disciplines, and we hope that this work will provide guidance to harness artificial intelligence power for simulation-based materials discovery.

## Conflicts of interest

There are no conflicts to declare.

## Supplementary Material

Supplementary information

## References

[cit1] Sholl D. S., Lively R. P. (2016). Nature.

[cit2] Brennecke J. F., Eckert C. A. (1989). AIChE J..

[cit3] Kattner U. R. (1997). JOM.

[cit4] Foo K. Y., Hameed B. H. (2010). Chem. Eng. J..

[cit5] Siepmann J. I., Brennecke J. F., Allan D. T., Klein M. T., Savage P. E., Schatz G. C., Winnik F. M. (2018). J. Chem. Eng. Data.

[cit6] Gmehling J., Constantinescu D., Schmid B. (2015). Annu. Rev. Chem. Biomol. Eng..

[cit7] Panagiotopoulos A. Z. (1992). Mol. Simul..

[cit8] Smit B., Maesen T. L. M. (2008). Chem. Rev..

[cit9] Duren T., Bae Y.-S., Snurr R. Q. (2009). Chem. Soc. Rev..

[cit10] Ruthven D. M., Principles of Adsorption and Adsorption Processes, Wiley, 1984.

[cit11] Wankat P. C., Separation Process Engineering: Includes Mass Transfer Analysis, Pearson Education, 2016.

[cit12] Krizhevsky A., Sutskever I. and Hinton G. E., Advances in Neural Information Processing Systems 25, 2012, pp. 1097–1105.

[cit13] He K., Zhang X., Ren S. and Sun J., 2016 IEEE Conference on Computer Vision and Pattern Recognition, CVPR, 2016, pp. 770–778.

[cit14] Mikolov T., Sutskever I., Chen K., Corrado G. S. and Dean J., Advances in Neural Information Processing Systems 26, 2013, pp. 3111–3119.

[cit15] Sutskever I., Vinyals O. and Le Q. V., Advances in Neural Information Processing Systems 27, 2014, pp. 3104–3112.

[cit16] Mnih V., Kavukcuoglu K., Silver D., Rusu A. A., Veness J., Bellemare M. G., Graves A., Riedmiller M., Fidjeland A. K., Ostrovski G., Petersen S., Beattie C., Sadik A., Antonoglou I., King H., Kumaran D., Wierstra D., Legg S., Hassabis D. (2015). Nature.

[cit17] Silver D., Schrittwieser J., Simonyan K., Antonoglou I., Huang A., Guez A., Hubert T., Baker L., Lai M., Bolton A., Chen Y., Lillicrap T., Hui F., Sifre L., van den Driessche G., Graepel T., Hassabis D. (2017). Nature.

[cit18] OpenAI, OpenAI Five, https://blog.openai.com/openai-five/, 2018, accessed September 11, 2018.

[cit19] Torlai G., Melko R. G. (2016). Phys. Rev. B.

[cit20] Ha M. Y., Yoon T. J., Tlusty T., Jho Y., Lee W. B. (2018). J. Phys. Chem. Lett..

[cit21] Schmitz J. E., Zemp R. J., Mendes M. J. (2006). Fluid Phase Equilib..

[cit22] Moraes J. E. F., Quina F. H., Nascimento C. A. O., Silva D. N., Chiavone-Filho O. (2004). Environ. Sci. Technol..

[cit23] Simon C. M., Mercado R., Schnell S. K., Smit B., Haranczyk M. (2015). Chem. Mater..

[cit24] Borboudakis G., Stergiannakos T., Frysali M., Klontzas E., Tsamardinos I., Froudakis G. E. (2017). npj Comput. Mater..

[cit25] Hinton G. E., Osindero S., Teh Y.-W. (2006). Neural Computation.

[cit26] Zhao J., Mathieu M. and LeCun Y., Energy-based Generative Adversarial Network, 2016, arXiv:1609.03126, arXiv.org e-Print archive, https://arxiv.org/abs/1609.03126.

[cit27] Berthelot D., Schumm T. and Metz L., BEGAN: Boundary Equilibrium Generative Adversarial Networks, 2017, arXiv:1703.10717, arXiv.org e-Print archive, https://arxiv.org/abs/1703.10717.

[cit28] Yosinski J., Clune J., Bengio Y. and Lipson H., Advances in Neural Information Processing Systems 27, 2014, pp. 3320–3328.

[cit29] Oquab M., Bottou L., Laptev I. and Sivic J., 2014 IEEE Conference on Computer Vision and Pattern Recognition, 2014, pp. 1717–1724.

[cit30] Howard J. and Ruder S., Universal Language Model Fine-tuning for Text Classification, 2018, arXiv:1801.06146, arXiv.org e-Print archive, https://arxiv.org/abs/1801.06146.

[cit31] Freundlich H. (1907). Z. Phys. Chem..

[cit32] Langmuir I. (1918). J. Am. Chem. Soc..

[cit33] Brunauer S., Emmett P. H., Teller E. (1938). J. Am. Chem. Soc..

[cit34] Sips R. (1948). J. Chem. Phys..

[cit35] Mathias P. M., Kumar R., Moyer J. D., Schork J. M., Srinivasan S. R., Auvil S. R., Talu O. (1996). Ind. Eng. Chem. Res..

[cit36] Khan A., Ataullah R., Al-Haddad A. (1997). J. Colloid Interface Sci..

[cit37] Toth J. (2000). J. Colloid Interface Sci..

[cit38] Cavenati S., Grande C. A., Rodrigues A. E. (2004). J. Chem. Eng. Data.

[cit39] Hefti M., Joss L., Bjelobrk Z., Mazzotti M. (2016). Faraday Discuss..

[cit40] Myers A. L., Prausnitz J. M. (1965). AIChE J..

[cit41] Suwanayuen S., Danner R. P. (1980). AIChE J..

[cit42] Ruthven D. M., Wong F. (1985). Ind. Eng. Chem. Fundam..

[cit43] Talu O., Zwiebel I. (1986). AIChE J..

[cit44] Richter E., Wilfried S., Myers A. L. (1989). Chem. Eng. Sci..

[cit45] Carsky M., Do D. (1999). Adsorption.

[cit46] Basu S., Henshaw P. F., Biswas N., Kwan H. K. (2010). Can. J. Chem. Eng..

[cit47] Turan N. G., Mesci B., Ozgonenel O. (2011). Chem. Eng. J..

[cit48] Morse G., Jones R., Thibault J., Tezel F. H. (2011). Adsorption.

[cit49] Menon V., Komarneni S. (1998). J. Porous Mater..

[cit50] Morris R., Wheatley P. (2008). Angew. Chem., Int. Ed..

[cit51] Primo A., Garcia H. (2014). Chem. Soc. Rev..

[cit52] Kosinov N., Gascon J., Kapteijn F., Hensen E. J. M. (2016). J. Membr. Sci..

[cit53] Bezouhanova C. P., Jabur F. A. (1993). React. Kinet. Catal. Lett..

[cit54] Aghaziarati M., Kazemeini M., Soltanieh M., Sahebdelfar S. (2007). Ind. Eng. Chem. Res..

[cit55] Rani V. R., Srinivas N., Kulkarni S. J., Raghavan K. V. (2002). J. Mol. Catal. A: Chem..

[cit56] Barnett K. J., McClelland D. J., Huber G. W. (2017). ACS Sustainable Chem. Eng..

[cit57] Li S., Tuan V. A., Falconer J. L., Noble R. D. (2001). Chem. Mater..

[cit58] Li S., Tuan V. A., Falconer J. L., Noble R. D. (2001). Ind. Eng. Chem. Res..

[cit59] Bai P., Jeon M. Y., Ren L., Knight C., Deem M. W., Tsapatsis M., Siepmann J. I. (2015). Nat. Commun..

[cit60] DeJaco R. F., Elyassi B., Dorneles de Mello M., Mittal N., Tsapatsis M., Siepmann J. I. (2018). J. Chem. Phys..

[cit61] Jin A., Li Y., Yang W. (2018). Ind. Eng. Chem. Res..

[cit62] Krishna R., van Baten J. M. (2010). Langmuir.

[cit63] Bai P., Tsapatsis M., Siepmann J. I. (2012). Langmuir.

[cit64] Oudshoorn A., van der Wielen L. A. M., Straathof A. J. J. (2009). Ind. Eng. Chem. Res..

[cit65] Liu J., Qi Y., Meng Z. Y., Fu L. (2017). Phys. Rev. B.

[cit66] Shen H., Liu J., Fu L. (2018). Phys. Rev. B.

[cit67] Desgranges C., Delhommelle J. (2018). J. Chem. Phys..

[cit68] Witman M., Mahynski N. A., Smit B. (2018). J. Chem. Theory Comput..

[cit69] Mnih A. and Gregor K., Proceedings of the 31st International Conference on International Conference on Machine Learning, vol. 32, 2014, pp. II-1791–II-1799.

[cit70] Kullback S., Leibler R. A. (1951). Ann. Math. Stat..

[cit71] Agueda V. I., Delgado J. A., Uguina M. A., Sotelo J. L., Garcia A. (2013). Sep. Purif. Technol..

[cit72] Gabruś E., Witkiewicz K., Nastaj J. (2018). Chem. Eng. J..

[cit73] DeJaco R. F., Bai P., Tsapatsis M., Siepmann J. I. (2016). Langmuir.

[cit74] Sarkisov L., Monson P. A. (2000). Langmuir.

[cit75] Clevert D.-A., Unterthiner T. and Hochreiter S., Fast and Accurate Deep Network Learning by Exponential Linear Units (ELUs), 2015, arXiv:1511.07289, arXiv.org e-Print archive, https://arxiv.org/abs/1511.07289.

[cit76] Baxter J. (1997). Machine Learning.

[cit77] Breiman L. (1996). Machine Learning.

[cit78] Carney J. G., Cunningham P. and Bhagwan U., IJCNN'99. International Joint Conference on Neural Networks, Proceedings (Cat. No. 99CH36339), 1999, vol. 2, pp. 1215–1218.

[cit79] Bai P., Tsapatsis M., Siepmann J. I. (2013). J. Phys. Chem. C.

[cit80] Cybenko G. (1989). Math. Control, Signals, Syst..

[cit81] Goodfellow I., Bengio Y. and Courville A., Deep Learning, MIT Press, 2016.

[cit82] Peng D.-Y., Robinson D. B. (1976). Ind. Eng. Chem. Fundam..

[cit83] Hinton G. E., Srivastava N., Krizhevsky A., Sutskever I. and Salakhutdinov R. R., Improving Neural Networks by Preventing Co-adaptation of Feature Detectors, 2012, arXiv:1207.0580, arXiv.org e-Print archive, https://arxiv.org/abs/1207.0580.

[cit84] Raissi M., Perdikaris P., Karniadakis G. (2019). J. Comp. Physiol..

