# Supplementary material for: Deep neural network learning of complex binary sorption equilibria from molecular simulation data
Source: Chem Sci. 2019 Mar 18;10(16):4377–88. doi: 10.1039/c8sc05340e (PMC6482883; doi:10.1039/c8sc05340e)
Supplement: Supplementary file 1 [file SC-010-C8SC05340E-s001.pdf]

Electronic Supplementary Material (ESI) for Chemical Science.
This journal is © The Royal Society of Chemistry 2019

Supplementary Information: Deep Neural Network Learning of Complex Binary Sorption Equilibria from Molecular Simulation Data

Yangzesheng Sun,[†] Robert F. DeJaco,[†,‡] and J. Ilja Siepmann[*,†,‡]

†*Department of Chemistry, University of Minnesota, Minneapolis, Minnesota 55455, United States*

‡*Department of Chemical Engineering and Materials Science, University of Minnesota, Minneapolis, Minnesota 55455, United States*

E-mail: siepmann@umn.edu

Phone: +1 (612) 624-1844. Fax: +1 (612) 626-7541

Contents

S1 Machine Learning Methods **S3**

S1.1 Neural Network Structures and Training S3

S1.2 Variable Transformations S3

S2 Simulation Details **S5**

S3 Isotherm Predictions by SorbNet **S9**

S4 Uncertainty Estimation by SorbNet Ensemble **S13**

S5 Desorption Temperature Optimization **S15**

References **S16**

S1 Machine Learning Methods

S1.1 Neural Network Structures and Training

The neural networks (SorbNet, p–v mapping network, dense network, shallow network) were implemented using the Keras[S1] (2.1.4) framework with TensorFlow[S2] (1.6.0) backend. Exponential linear unit (ELU) activation functions[S3] were used throughout the hidden layers of all neural networks, and the sigmoid activation function was used on the output layer in neural networks predicting the equilibrium sorption loading. The p–v mapping network does not have an activation function on the output layer. Weight matrices of all neural networks were initialized using the Xavier initialization method[S4] and biases were initialized as zeros. The neural networks were trained using the Adam optimizer[S5] at an initial learning rate of 0.002 and an exponential decay of 0.002 per epoch. The binary cross-entropy loss function was used to train networks for sorption loading prediction, and the mean square error loss was used to train the p–v network. No regularization algorithm (weight decay, dropout, etc.) was applied in the training process. Except for the pre-training for only 200 epochs in transfer learning applications on temperature-dependence prediction, each neural network was trained for 500 epochs on the simulation data of a sorption system.

The neural network codes and dataset are available at
`https://github.com/SiepmannGroup/desorption`
and
`https://github.com/SiepmannGroup/MCCCS_DB/tree/master/diol-desorption`
respectively.

S1.2 Variable Transformations

Variable transformations were performed when training the neural network. The volumes selected to perform the simulations were uniformly distributed in logarithmic space, therefore the relative volume v as the vapor volume per zeolite unit cell was transformed into $-\log_{10} v$ in the training

process. Following chemical intuition, inverse temperatures instead of absolute temperatures were used as the training feature. The transformation of T to $1000/T$ was used to make all training features $(n_1, n_2, -\log_{10} v, 1000/T)$ distribute in similar numerical ranges and to agree with engineering conventions in representing temperature dependence. Preliminary training results showed that using inverse temperature resulted in a lower error compared with directly scaling the temperature, and further shifting or scaling [e.g., zero-mean or normalize to (0, 1)] of the features did not substantially improve the model performance. Hence, the transformed training features $(n_1, n_2, -\log_{10} v, 1000/T)$ were used without further modifications.

S2 Simulation Details

Monte Carlo simulations were performed here in the isochoric–isothermal version of the Gibbs ensemble[S6,S7] using a set-up with two simulation boxes. These simulations model the desorptive drying stage in the separation of a diol/water using an adsorption–drying–desorption process. An illustration of such a process for the MFI-C5-W system is shown in Figure S1.

The TIP4P model for water[S8] and the united-atom version of the TraPPE force field for ethanol, butane-1,4-diol, and pentane-1,5-diol,[S9,S10] and the TraPPE-zeo force field for all-silica zeolite framework atoms[S11] were used. Following the TraPPE–UA force field for ethers and glycols,[S12] intramolecular Coulombic interactions between pseudo-atoms separated by three bonds (for α- and ω-CH_2 groups in 1,4-butanediol) were scaled by a factor of $1/2$. The Coulomb and Lennard-Jones interactions of sorbate molecules with the rigid zeolite framework were determined using pretabulation and interpolation[S13,S14] with a grid spacing of 0.1 Å. The vapor phase was treated as a real gas with a cutoff distance at $L/2$ and a volume of L^3, while the cutoff distance in each zeolite phase was 14.0 Å. Analytical tail corrections[S15] were used to estimate Lennard-Jones interactions beyond these cutoff distances. Coulomb interactions were described using the Ewald summation method[S15] with a screening parameter of $\kappa = 3.2/r_{\text{cut}}$ and an upper bound of the reciprocal space summation at $K_{\text{max}} = \lceil \kappa L_{\text{box}} \rceil$.

This work considered the all-silica forms of zeolites MFI and LTA using the structures determined by van Konigsveld *et al.*[S16] and by Boal *et al.*,[S17] respectively. The MFI and LTA zeolites were treated as rigid frameworks. The unit cell parameters and number of replications in **a**, **b**, and **c** used to obtain simulation boxes with linear dimensions larger than 28.0 Å are presented in Table S1.

Table S1: Structures of All-Silica Zeolites

Framework	**a** [Å]	**b** [Å]	**c** [Å]	α [°]	β [°]	γ [°]	Replications $\mathbf{a} \times \mathbf{b} \times \mathbf{c}$
MFI	20.022	19.899	13.383	90.000	90.000	90.000	$2 \times 2 \times 3$
LTA	11.857	11.857	11.857	90.000	90.000	90.000	$3 \times 3 \times 3$

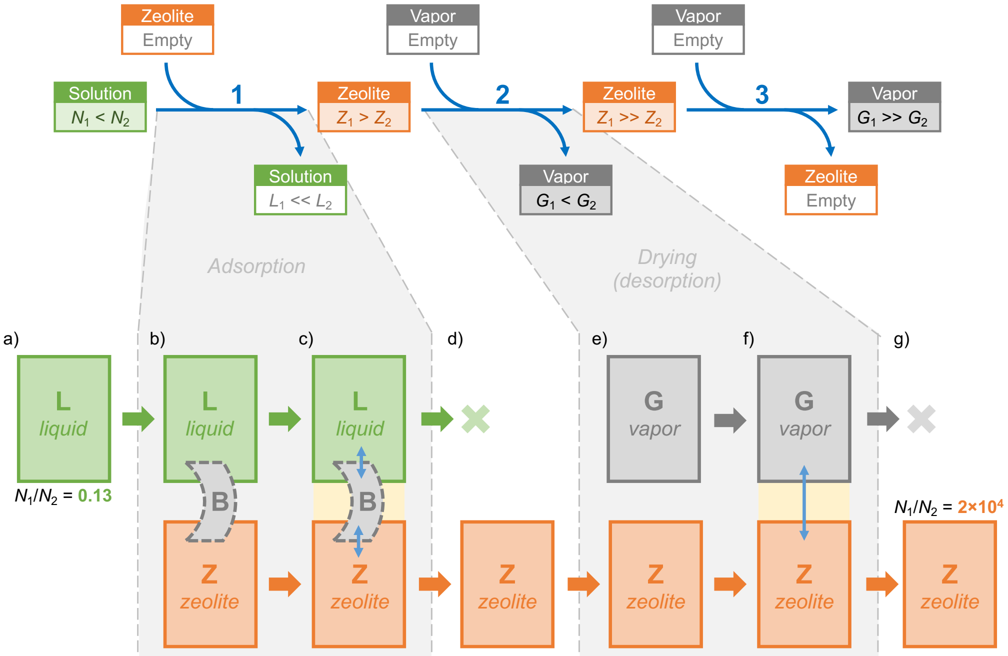

T (K)	323	323	323	323	403	403	403
Z_1	/	0	95.996 ± 0.003	96	96	93.7 ± 0.4	93
Z_2	/	0	9.6 ± 1.3	10	10	0.004 ± 0.004	0
B_1	/	0	17.0 ± 1.8	/	/	/	/
B_2	/	0	31 ± 3	/	/	/	/
L_1	128	128	15.0 ± 1.8	/	/	/	/
L_2	968	968	927 ± 2	/	/	/	/
G_1	/	/	/	/	0	2.3 ± 0.4	/
G_2	/	/	/	/	0	9.996 ± 0.004	/
$\mathbf{N_1}$	**128**	**128**	**128**	**96**	**96**	**96**	**93**
$\mathbf{N_2}$	**968**	**968**	**968**	**10**	**10**	**10**	**0**

Figure S1: Adsorptive separation of a 1,5-pentanediol (1) / water (2) mixture in the MFI zeolite. The top part shows a schematic for the three-stage separation process of a diol/solvent mixture. The first two stages are of interest here because they involve mixtures, whereas the final desorption stage involves essentially only the diol. The middle part shows the GEMC simulation setups for the solution-phase adsorption stage and the desorptive drying stage: (a) an aqueous solution of diol is represented by a liquid simulation box **L**. (b) **L** is coupled with an empty zeolite simulation box **Z** to simulate the adsorption from the solution. A vapor transfer medium **B** is used to facilitate particle exchange between solution-phase and zeolite. (c) The solution phase has reached equilibrium with the zeolite phase where the zeolite is highly selective for the diol. (d) **L** and **B** are removed, leaving **Z** loaded with both diol and water. (e) An empty vapor simulation box **G** is coupled with **Z** to simulate the desorption into a gas phase. (f) The vapor phase has reached equilibrium with the zeolite phase during which water is selectively desorbed. (g) **G** is removed, leaving **Z** with extremely enriched diol. Light yellow stripes among simulation boxes indicate the establishment of equilibrium. Double-headed blue arrows denote particle exchanges between two simulation boxes. The table at the bottom provides information on the numbers of molecules found in the individual simulation boxes and in the entire system (N) where subscripts 1 and 2 denote diol (1) and water (2), respectively. Dimensions of **Z** are given in Table S1. For the example shown here, the average box lengths of **L** and **B** in the adsorption simulation were 31.47 ± 0.13 Å and 6727.289 ± 0.014 Å, respectively. The volume of **B** was maintained through incorporation of 6917465 non-interacting particles. The box length of **G** in the desorption simulations was fixed at 1024 Å.

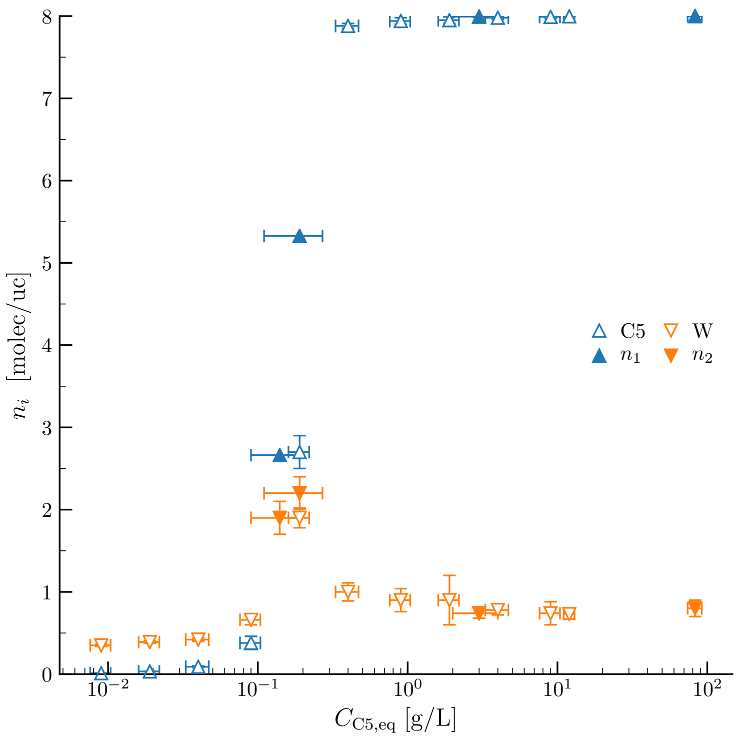

Figure S2: Solution-phase adsorption isotherms of C5 (1) and W (2) in the MFI-C5-W system (data taken from DeJaco *et al.*[S18]). Filled symbols denote the initial loadings used in the desorptive drying simulations listed in Table S2.

For each of the four zeolite-diol-solvent system, four different compositions (numbers of diol and solvent molecules) were investigated that correspond to zeolite loadings observed during solution-phase adsorption simulations.[S18] The solution-phase adsorption isotherms and initial loadings for vapor-phase desorption in the MFI-C5-W system are illustrated in Figure S2. The simulations in the present work were started with all diol and solvent molecules in the zeolite phase, and information on these loadings is summarized in Table S2. For each of these 16 system/composition combinations, 256 temperature/volume combinations were investigated as follows: a set of 16 temperatures (T = 343, 353, 363, 373, 383, 393, 403, 413, 423, 433, 443, 453, 463, 473, 483, and 493 K) and a set of 16 vapor-phase boxlengths (L = 24, 48, 64, 128, 256, 512, 1024, 2048, 4096, 8192, 16384, 32768, 65536, 131072, 262144, and 524288 Å).

Table S2: State Points used for Adsorption Simulations, Numbers of Molecules in the System and Per Unit Cell and Lengths of Production Periods for the Desorptive Drying Simulations

	Adsorption[a]	Desorptive Drying				
Zeolite-Diol-Solvent	C_{ads} [g/mL]	N_{diol}	$N_{solvent}$	n_{diol}	$n_{solvent}$	10^3 MCCs
MFI-C5-W	0.083 ± 0.009	96	10	8.00	0.83	21–50
MFI-C5-W	0.0033 ± 0.0010	95	11	7.92	0.92	21–50
MFI-C5-W	0.00019 ± 0.00003	64	25	5.33	2.08	24–68
MFI-C5-W	0.00014 ± 0.00005	32	23	2.67	1.92	42–100
MFI-C4-W	0.4731 ± 0.0014	105	16	8.75	1.33	20–47
MFI-C4-W	0.041 ± 0.005	105	13	8.75	1.08	21–50
MFI-C4-W	0.009 ± 0.002	95	29	7.92	2.42	19–48
MFI-C4-W	0.0033 ± 0.0009	60	30	5.00	2.50	27–76
MFI-C5-E	0.647 ± 0.011	84	23	7.00	1.92	22–49
MFI-C5-E	0.559 ± 0.014	66	56	5.50	4.67	19–45
MFI-C5-E	0.37 ± 0.02	61	66	5.08	5.50	19–45
MFI-C5-E	0.197 ± 0.011	41	98	3.42	8.17	19–46
LTA-C5-W	0.420 ± 0.007	75	34	2.78	1.26	18–54
LTA-C5-W	0.214 ± 0.009	72	42	2.67	1.56	18–52
LTA-C5-W	0.085 ± 0.011	58	77	2.15	2.85	16–48
LTA-C5-W	0.024 ± 0.003	56	77	2.07	2.85	16–50

[a] All adsorption state points were conducted at $T_{ads} = 323$ K and $p_{ads} = 1.0$ bar.

For all simulations, the set of trial moves included rigid-body translations and rotations around the center-of-mass for all sorbate molecules, and coupled-decoupled configurational-bias Monte Carlo (CD-CBMC) moves[S19] for all sorbate types except water. CD-CBMC strategies were also employed for particle transfer moves between the two phases.[S19,S20] A total of 32 independent simulations were used at each state point investigated. All simulations were wrapped in a job submission on *Mira*, a leadership-class supercomputer at Argonne National Laboratory. The equilibration period consisted of a 6 h job followed by a 12 h job. The production period consisted of two sequential 12 h jobs. The number of Monte Carlo cycles (where a cycle consists of $N_{diol} + N_{solvent}$ randomly selected moves) completed during the 24 h production period ranged from 16000 to 100000 depending on the total number of diol and solvent molecules, the number of interaction sites (4 for water and ethanol, 8 for 1,4-butanediol, and 9 for 1,5-pentanediol), and the distribution of these molecules over the two simulation boxes. The specific ranges for each of the 16 system/composition combinations are provided in Table S2.

S3 Isotherm Predictions by SorbNet

Figures S3–S5 show the SorbNet predictions and isotherm curves for the other three sorption systems (MFI-C4-W, MFI-C5-E, LTA-C5-W). For all sorption systems, a sigmoid-like isotherm shape was observed for the diol, and an increasing loading with a low-volume onset was observed for the solvents.

At the smallest volume and the highest temperature, the alkanediol loading shows a slight decrease instead of reaching saturation (see Figures S4 and S5). This erroneous result can be attributed to the partial condensation in the reservoir phase when the number density approaches 2 molecules per nm^3. The droplet formation lowers the chemical potentials of diol and solvent in the spinodally decomposed reservoir phase. As a result, the loading in the zeolite phase is decreased.

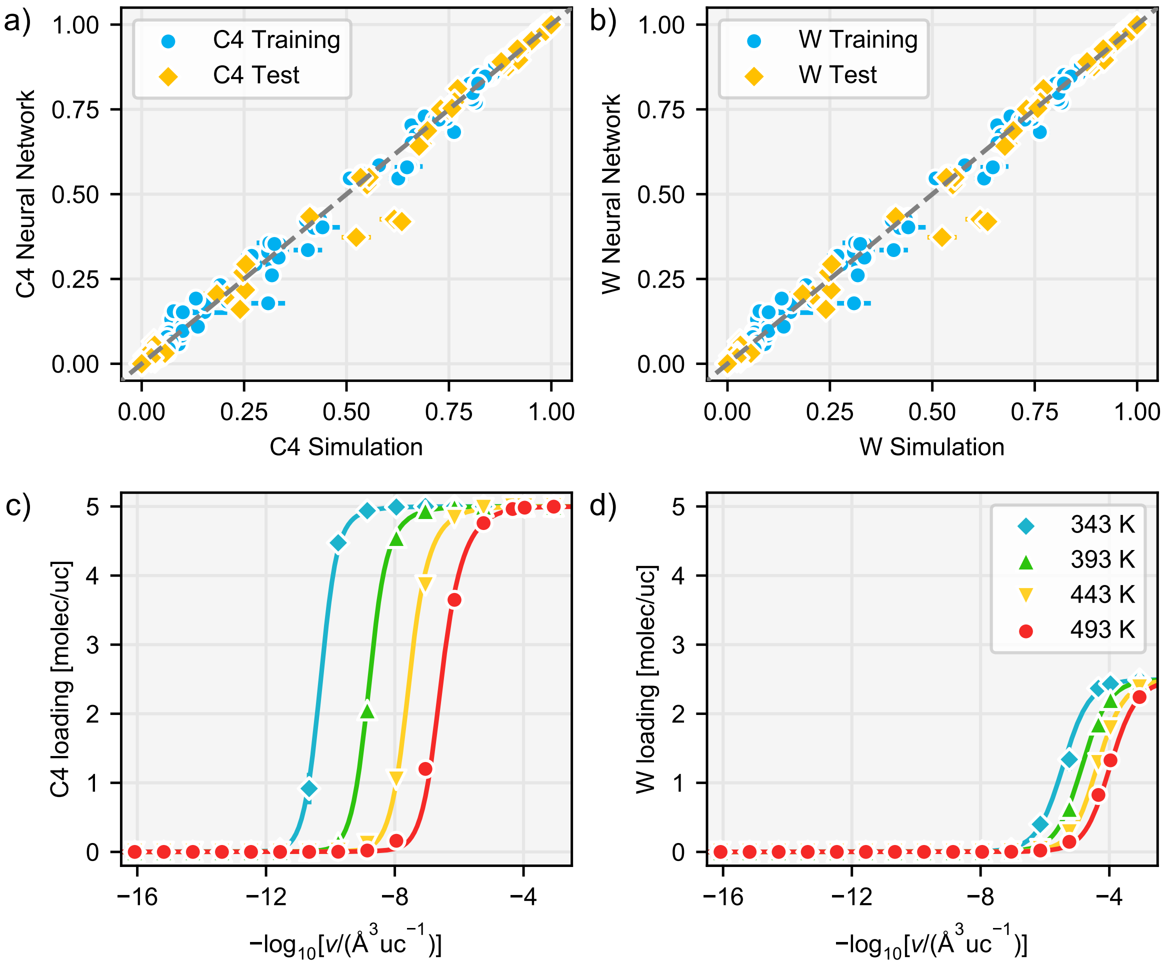

Figure S3: (a-b) Scatter plot of SorbNet predictions of C4 (a) and W (b) fractional loadings for training and test temperatures ($\hat{y}_i$) versus fractional loadings obtained from simulations (y_i) in the MFI-C4-W system. (c-d) Loading–volume sorption isotherms for 1,4-butanediol (c) and water (d) in the MFI-C4-W system at test set temperatures and an initial sorbate loading of C4:W = 5.00:2.50 (molec/uc). Symbols denote simulation data and lines denote neural network predictions.

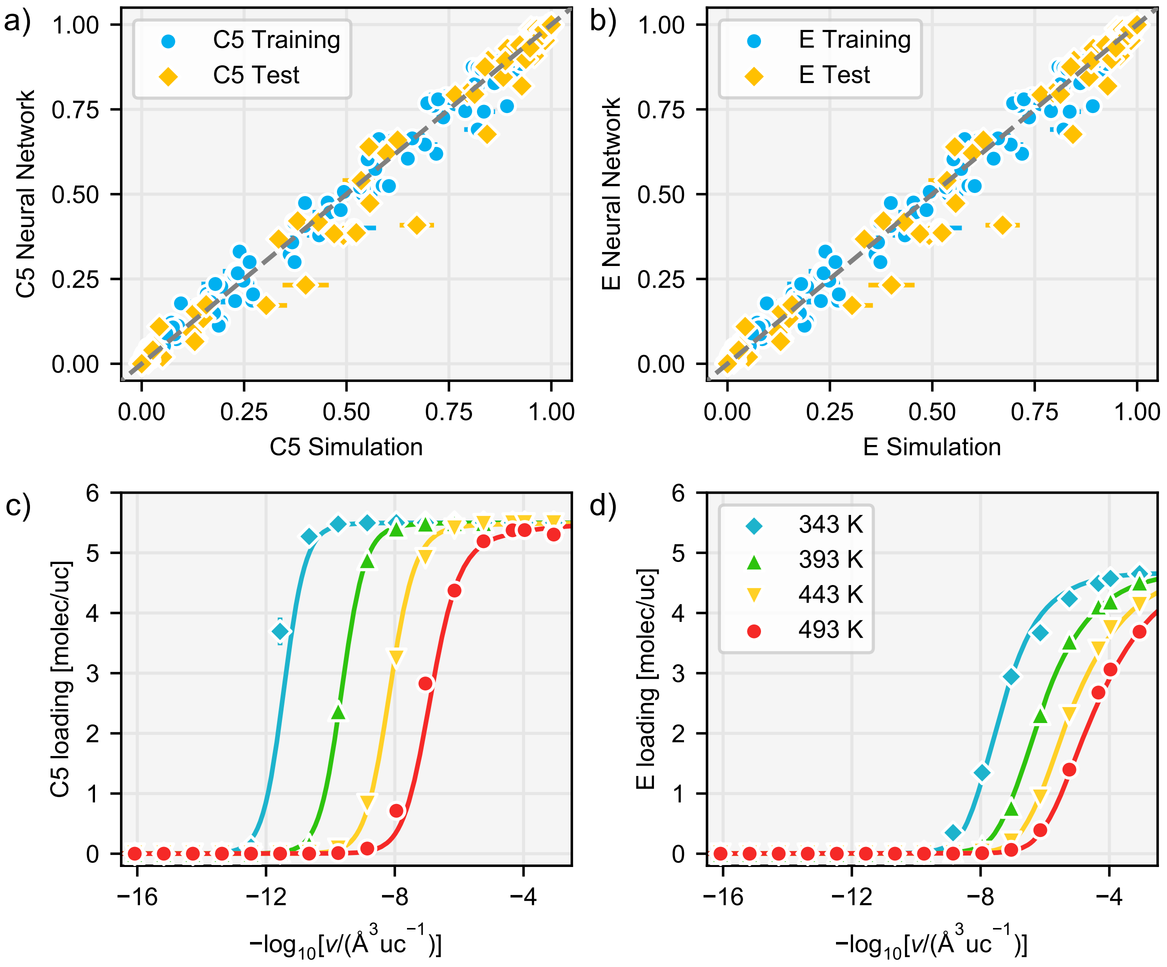

Figure S4: (a-b) Scatter plot of SorbNet predictions of C5 (a) and E (b) fractional loadings for training and test temperatures ($\hat{y}_i$) versus fractional loadings obtained from simulations (y_i) in the MFI-C5-E system. (c-d) Loading–volume sorption isotherms for 1,5-pentanediol (c) and ethanol (d) in the MFI-C5-E system at test set temperatures and an initial sorbate loading of C5:E = 5.50:4.67 (molec/uc). Symbols denote simulation data and lines denote neural network predictions.

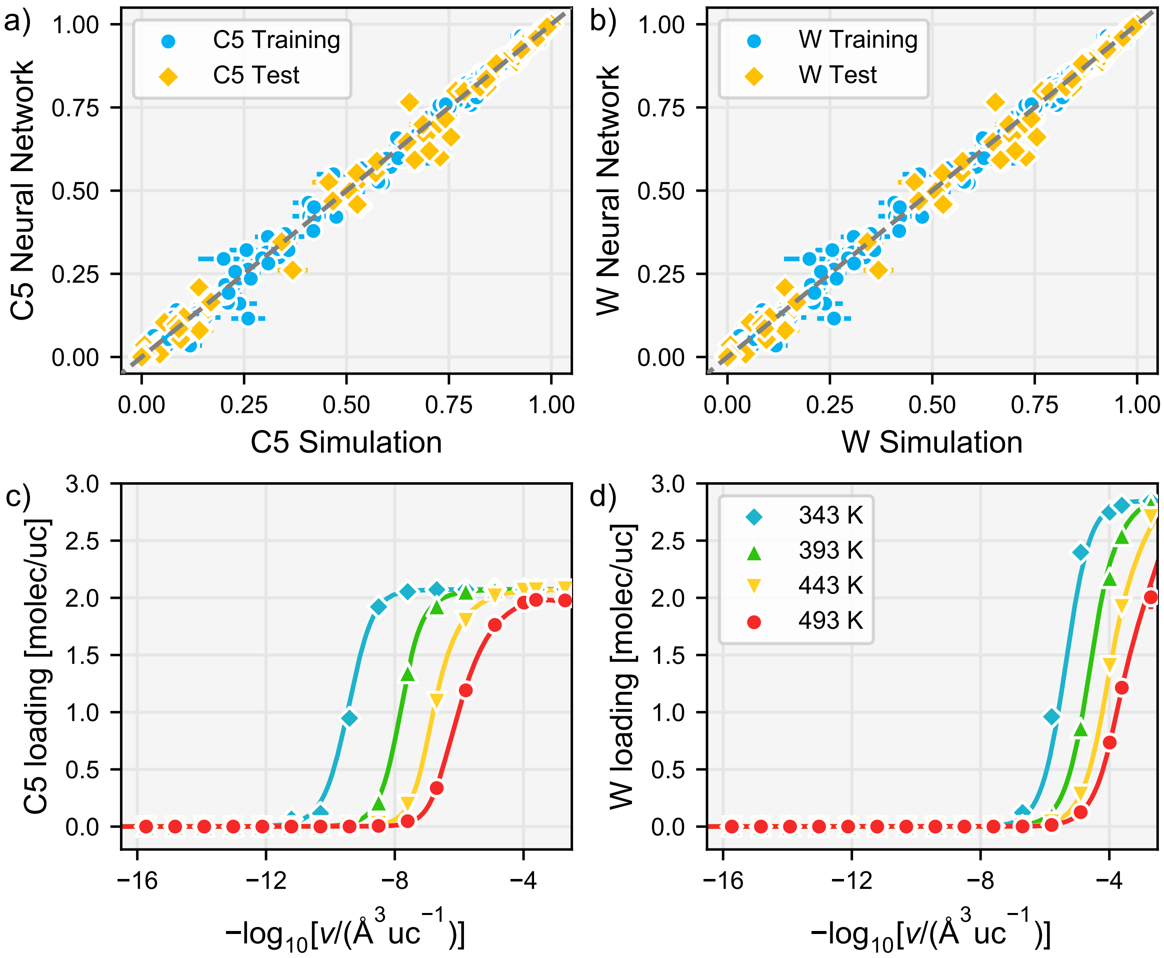

Figure S5: (a-b) Scatter plot of SorbNet predictions of C5 (a) and W (b) fractional loadings for training and test temperatures ($\hat{y}_i$) versus fractional loadings obtained from simulations (y_i) in the LTA-C5-W system. (c-d) Loading–volume sorption isotherms for 1,5-pentanediol (c) and water (d) in the LTA-C5-W system at test set temperatures and an initial sorbate loading of C5:W = 2.07:2.85 (molec/uc). Symbols denote simulation data and lines denote neural network predictions.

S4 Uncertainty Estimation by SorbNet Ensemble

The information obtained from multiple independent simulations can be utilized to train an ensemble of multiple neural networks for uncertainty estimation. To be distinguished from the statistical mechanical ensemble in molecular simulation, *ensemble* in this section refers to a collection of multiple machine learning models.[S21] In the present work, ensemble learning on independent simulation data was carried out by dividing the full simulation dataset into 32 distinct subsets, then training 32 SorbNets each on a subset of the simulation data. Each subset of simulation data contained one independent simulation at all 1024 state points, and predictions by the SorbNet ensemble were obtained by averaging 32 individual outputs of the neural networks. The SorbNet ensemble is also able to estimate the uncertainty of its predictions as the standard deviation of 32 individual outputs. Trained on the MFI-C5-W system, the SorbNet ensemble uncertainty qualitatively agrees with the simulation uncertainty which is highest when loading changes rapidly with log-volume (see Figure S6a) due to break-up of the cooperative hydrogen-bonding network formed in particular by 1,5-pentanediol in MFI at high loading (e.g., near 8 molecules per unit cell).[S18] In addition, the prediction of water loading becomes less confident when extrapolating to smaller vapor volumes as the neural networks do not see evident saturation of water at $T = 343$ K (see Figure S6b). Therefore, performing multiple simulations not only improves the accuracy of simulation results within the given walltime, but also provides machine learning with the potential to estimate uncertainties of the predictions.

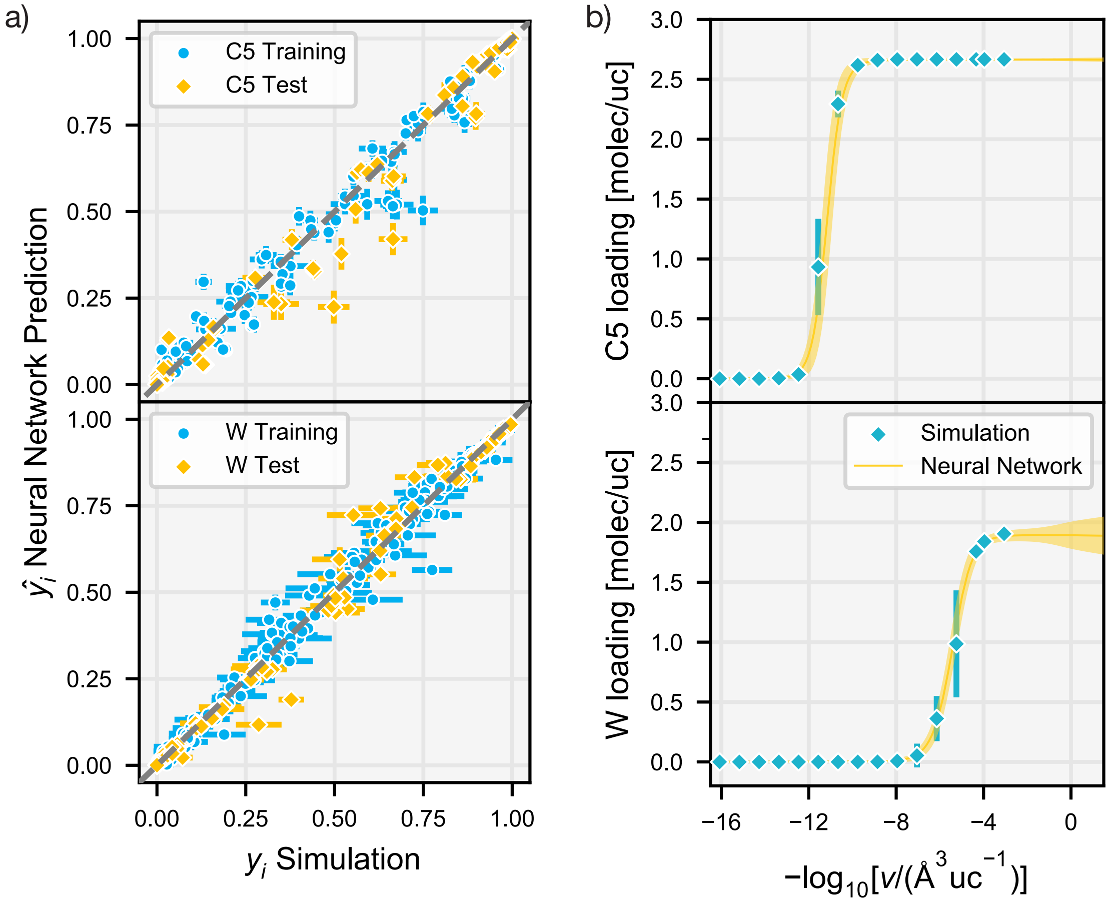

Figure S6: (a) Scatter plot with uncertainty estimation of SorbNet predictions for C5 and W fractional loadings for training and test temperatures versus fractional loadings obtained from simulations for the MFI-C5-W system. Horizontal error bars denote the standard deviation from 32 independent simulations and vertical error bars denote the standard deviation predicted by the SorbNet ensemble. (b) Loading–volume sorption isotherms with uncertainty estimation of the MFI-C5-W system at T = 343 K and an initial loading of C5:W = 2.67:1.92 molec/uc. Symbols with error bars denote simulation data and lines with shaded region denote predictions by the SorbNet ensemble. Error bars and shaded regions correspond to 3 times the standard deviation to better visualize uncertainties.

S5 Desorption Temperature Optimization

The “ground-truth” values of the vapor pressures were calculated from the total number density in the vapor-phase simulation box assuming ideal gas behavior. Due to the large additional computational cost in Monte Carlo simulations, direct calculations of the vapor pressure using the virial equation[S15] throughout the simulations was not conducted. In the pressure calculation and subsequent prediction tasks, simulation results corresponding to less than 0.1 molecules in the vapor-phase simulation box were removed to minimize the effects of large relative uncertainties. Another neural network to map pressure from vapor volume was used instead of directly training the SorbNet on the pressure variable. This is because the already trained SorbNet has encoded information about the sorption system, and directly using pressure to predict sorption loading worked much more poorly as simulations were not conditioned on the vapor pressure. After generating the loading surface and isobaric adsorption curves, the optimal temperature is found by a linear search in 0.2 K intervals for the maximum diol/solvent molar ratio in the zeolite phase with the constraint of the adsorbed diol mole fraction being no smaller than 99%. The pressure dependence of the optimal temperature was obtained by performing searches between 10^{-3} kPa and 10^{2} kPa in the logarithmic space. Since neural networks are trained in a gradient-based nature, the brute-force search method employed in this work can be readily improved by using gradient information produced by neural network training to optimize any operation condition.

References

(S1) Chollet, F. Keras. https://github.com/fchollet/keras, 2015.

(S2) Abadi, M.; Barham, P.; Chen, J.; Chen, Z.; Davis, A.; Dean, J.; Devin, M.; Ghemawat, S.; Irving, G.; Isard, M.; Kudlur, M.; Levenberg, J.; R.Monga,; Moore, S.; Murray, D. G.; Steiner, B.; Tucker, P.; Vasudevan, V.; Warden, P.; Wicke, M.; Yu, Y.; Zheng, X. TensorFlow: A System for Large-Scale Machine Learning. 12th USENIX Symposium on Operating Systems Design and Implementation (OSDI 16). 2016; pp 265–283.

(S3) Clevert, D.-A.; Unterthiner, T.; Hochreiter, S. Fast and Accurate Deep Network Learning by Exponential Linear Units (ELUs). 2015; arXiv:1511.07289. arXiv.org e-Print archive. https://arxiv.org/abs/1511.07289.

(S4) Glorot, X.; Bengio, Y. Understanding the difficulty of training deep feedforward neural networks. In Proceedings of the International Conference on Artificial Intelligence and Statistics (AISTATS'10). 2010.

(S5) Kingma, D. P.; Ba, J. Adam: A Method for Stochastic Optimization. 2014; arXiv:1412.6980. arXiv.org e-Print archive. https://arxiv.org/abs/1412.6980.

(S6) Panagiotopoulos, A. Z. Direct Determination of Phase Coexistence Properties of Fluids by Monte-Carlo Simulation in a New Ensemble. *Mol. Phys.* **1987**, *61*, 813–826.

(S7) Panagiotopoulos, A. Z.; Quirke, N.; Stapleton, M.; Tildesley, D. J. Phase Equilibria by Simulation in the Gibbs Ensemble - Alternative Derivation, Generalization and Application to Mixture and Membrane Equilibria. *Mol. Phys.* **1988**, *63*, 527–545.

(S8) Jorgensen, W. L.; Chandrasekhar, J.; Madura, J. D.; Impey, R. W.; Klein, M. L. Comparison of Simple Potential Functions for Simulating Liquid Water. *J. Chem. Phys.* **1983**, *79*, 926–935.

(S9) Martin, M. G.; Siepmann, J. I. Transferable Potentials for Phase Equilibria. 1. United-Atom Description of *n*-Alkanes. *J. Phys. Chem. B* **1998**, *102*, 2569–2577.

(S10) Chen, B.; Potoff, J. J.; Siepmann, J. I. Monte Carlo Calculations for Alcohols and Their Mixtures with Alkanes. Transferable Potentials for Phase Equilibria. 5. United-Atom Description of Primary, Secondary, and Tertiary Alcohols. *J. Phys. Chem. B* **2001**, *105*, 3093–3104.

(S11) Bai, P.; Tsapatsis, M.; Siepmann, J. I. TraPPE-Zeo: Transferable Potentials for Phase Equilibria Force Field for All-Silica Zeolites. *J. Phys. Chem. C.* **2013**, *117*, 24375–24387.

(S12) Stubbs, J. M.; Potoff, J. J.; Siepmann, J. I. Transferable Potentials for Phase Equilibria. 6. United-Atom Description for Ethers, Glycols, Ketones, and Aldehydes. *J. Phys. Chem. B.* **2004**, *108*, 17596–17605.

(S13) June, R. L.; Bell, A. T.; Theodorou, D. N. Prediction of Low Occupancy Sorption of Alkanes in Silicalite. *J. Phys. Chem.* **1990**, *94*, 1508–1516.

(S14) Bai, P.; Jeon, M. Y.; Ren, L.; Knight, C.; Deem, M. W.; Tsapatsis, M.; Siepmann, J. I. Discovery of Optimal Zeolites for Challenging Separations and Chemical Transformations using Predictive Materials Modeling. *Nat. Commun.* **2015**, *6*, 5912.

(S15) Allen, M. P.; Tildesley, D. J. *Computer Simulation of Liquids*; Oxford University Press: New York, 1987.

(S16) van Koningsveld, H.; van Bekkum, H.; Jansen, J. C. On the Location and Disorder of the Tetrapropylammonium (TPA) Ion in Zeolite ZSM-5 With Improved Framework Accuracy. *Acta Crystallogr. B.* **1987**, *43*, 127–132.

(S17) Boal, B. W.; Schmidt, J. E.; Deimund, M. A.; Deem., M. W.; Henling, L. M.; Brand, S. K.; Zones, S. I.; Davis, M. E. Facile Synthesis and Catalysis of Pure-Silica and Heteroatom LTA. *Chem. Mater.* **2015**, *27*, 7774–7779.

(S18) DeJaco, R. F.; Elyassi, B.; Dorneles de Mello, M.; Mittal, N.; Tsapatsis, M.; Siepmann, J. I. Understanding the Unique Sorption of Alkane-α,ω-diols in Silicalite-1. *J. Chem. Phys.* **2018**, *149*, 072331.

(S19) Martin, M. G.; Siepmann, J. I. Novel Configurational-Bias Monte Carlo Method for Branched Molecules. Transferable Potentials for Phase Equilibria. 2. United-Atom Description of Branched Alkanes. *J. Phys. Chemi. B* **1999**, *103*, 4508–4517.

(S20) Mooij, G. C. A. M.; Frenkel, D.; Smit, B. Direct Simulation of Phase Equilibria of Chain Molecules. *J. Phys.: Condens. Matter* **1992**, *4*, L225–L259.

(S21) Carney, J. G.; Cunningham, P.; Bhagwan, U. Confidence and Prediction Intervals for Neural Network Ensembles. IJCNN'99. International Joint Conference on Neural Networks. Proceedings (Cat. No.99CH36339). 1999; pp 1215–1218 vol.2.
